# Nada sobre as Mulheres, sem as Mulheres, é para as Mulheres

**DOI:** 10.36660/abc.20250323

**Published:** 2025-06-26

**Authors:** Camila Perez de Souza Arthur, Omar Asdrúbal Vilca Mejia

**Affiliations:** 1 Beneficiência Portuguesa de São Paulo São Paulo SP Brasil Beneficiência Portuguesa de São Paulo, São Paulo, SP - Brasil; 2 Instituto do Coração do Hospital das Clinicas da Faculdade de Medicina da Universidade de São Paulo São Paulo SP Brasil Instituto do Coração do Hospital das Clinicas da Faculdade de Medicina da Universidade de São Paulo, São Paulo, SP - Brasil

**Keywords:** Avaliação de Resultados em Cuidados de Saúde, Procedimentos Cirúrgicos Cardiovasculares, Saúde da Mulher

Análises ajustadas mostram que mulheres tem maior mortalidade do que os homens após cirurgia de revascularização miocárdica (CRM), além de receber uma técnica cirúrgica de menor qualidade.^1,2^ Estratégias atuais focam na otimização pré-operatória e na padronização dos cuidados intra e pós-operatórios, assim como de incluir mais mulheres nas pesquisas. Atualmente, mulheres são sub-representadas, chegando até 16% da amostra em alguns *trials.*^3^ Isto reforça o chamado por mais equidade e uma maior inclusão de mulheres nos projetos de pesquisa ou do contrário a realização de estudos específicos em mulheres.

Acredito que demoramos para refletir sobre esta realidade já que o sexo feminino é considerado preditor de mortalidade na CRM desde a publicação do CASS em 1981^4^ e se manteve constante, inclusive no nosso cenário como parte do InsCor.^5^ Isto foi novamente reconhecido neste importante artigo de Goncharov et al.^6^ Identificando o sexo feminino como preditor de mortalidade em um estudo unicêntrico e retrospectivo com 9.845 pacientes onde menos de 20% foram mulheres. No entanto ao contrário de outros estudos, a diferença de mortalidade entre mulheres e homens foi perdida após o pareamento.^6^

O *propensity score* ajusta uma análise com base nas variáveis observadas, enquanto estudos randomizados balanceiam tanto sobre variáveis conhecidas como desconhecidas. O viés surge quando diferenças nos resultados possam ser consequência de características secundárias dos pacientes e isto pode ser notório em uma amostra de quase 3 décadas.^6^ Dentro disto, talvez a variável que mais pode ter influenciado estes resultados foi a inclusão de cirurgias combinadas com CRM e não somente CRM isolada. Mulheres pelo geral são operadas apresentando mais comorbidades e chegam num estádio mais avançado para cirurgia.

Este é um achado global e que pode estar relacionado ao avanço silencioso da cardiopatia nas mulheres ou até ter um componente comportamental e cultural, o que justifica o pareamento dos grupos. Mas, independentemente de Goncharov ter mostrado que após o pareamento em um banco de dados unicêntrico retrospectivo não houve diferença na mortalidade entre mulheres e homens, ele conclui que o sexo feminino é sim um preditor independente de mortalidade após CRM.^6^ Um preditor que carrega a influência de outras variáveis conhecidas e desconhecidas da nossa prática diária e que devemos elucidar com urgência.^1,7,8^

Assim, por exemplo, dados mostram que nas CRM, mesmo mulheres apresentando menor tempo de cirurgia, de circulação extracorpórea, de anoxia, assim como um menor uso de enxertos arteriais e menos anastomoses distais, elas evoluem com mais complicações pós-operatórias imediatas. Isto nos faz refletir que não devemos somente padronizar cuidados iguais, mas que deveríamos avançar para cuidados cirúrgicos personalizados e otimizados em mulheres, tanto nos critérios diagnósticos, quanto no preparo, e o melhor momento para a intervenção, assim como nas melhores estratégias cirúrgicas para cada paciente.^9^

Personalizar é importante, existem fatores de risco que realmente são irreversíveis nas mulheres, como são uma menor constituição física, assim como um menor diâmetro e maior tortuosidade das coronárias, além de uma maior prevalência de fragilidade. As variações hormonais tornam mais grave a doença aterosclerótica no sexo feminino. Evidências recentes mostram que o tamanho físico menor nas mulheres leva a uma maior hemodiluição e taxas de hematócrito mais baixas durante a circulação extracorpórea que justifica, a necessidade de níveis mais liberais de transfusão em relação aos homens.^9,10^

Uma das áreas que mais tem nos preocupado está relacionada ao momento da indicação cirúrgica e ao preparo das pacientes mulheres, pois as recomendações vêm de diretrizes com número inexpressivo de mulheres.^11^ Portanto, nós estamos liberando pacientes mulheres para cirurgia com os critérios de pacientes homens, isto significa, com os mesmos níveis de hemoglobina sérica, hemoglobina glicosilada, função renal, albumina, etc. o que poderia explicar a diferença nos resultados. Isto amplia nosso horizonte, não só pela busca de como indicar melhor se não de como preparar, tratar e cuidar melhor das mulheres.^12^

**Figura 1:**
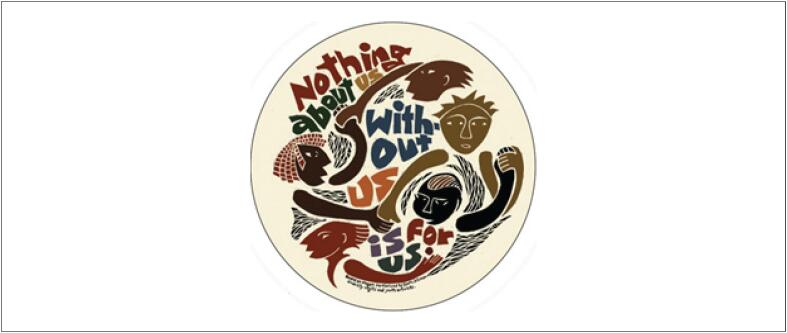
Nada sobre nós sem nós é para nós. Ricardo Levins Morales, 2010. Digital collections Joseph P. Healey Library.

Goncharov et al. estão de parabéns por conseguir trazer mais discussão sobre os resultados da CRM em mulheres, o que reforça o "*Call to action*" por mais evidências científicas incluindo mulheres para poder ser utilizadas efetivamente em mulheres.^6^ Promover um melhor atendimento para as mulheres se torna uma necessidade social que traz benefícios não somente por ser a população que mais cresce no mundo, se não por promover o princípio da inclusão e nos adequar às necessidades específicas que vão ao encontro com as políticas atuais. A frase "Nada sobre nós sem nós é para nos" baseia-se no princípio de participação, é usado para comunicar a ideia de que nenhuma política deve ser decidida sem a participação plena e direta dos membros do(s) grupo(s) afetado(s) por ela (Figura 1).^13,14^
