# Supplementary material for: Nada sobre as Mulheres, sem as Mulheres, é para as Mulheres
Source: Arq Bras Cardiol. 2025 Jun 26;122(5):e20250323. [Article in Portuguese] doi: 10.36660/abc.20250323 (PMC12269896; doi:10.36660/abc.20250323)
Supplement: Translation

# Translation: Nothing about Women without Women is for Women

**DOI:** 10.36660/abc.20250323i

**Authors:** Camila Perez de Souza Arthur, Omar Asdrúbal Vilca Mejia

**Affiliations:** 1 São Paulo SP Brazil Beneficiência Portuguesa de São Paulo, São Paulo, SP – Brazil; 2 São Paulo SP Brazil Instituto do Coração do Hospital das Clinicas da Faculdade de Medicina da Universidade de São Paulo, São Paulo, SP – Brazil

**Keywords:** Outcomes Assessment in Health Care, Cardiovascular Surgical Procedures, Women's Health

Adjusted analyses show that women have higher mortality rates than men after coronary artery bypass grafting (CABG), in addition to receiving a lower-quality surgical technique.^1,2^ Current strategies focus on preoperative optimization and standardization of intra- and postoperative care, as well as including more women in research. Currently, women are underrepresented, reaching up to 16% of the sample in some trials.^3^ This reinforces the call for greater equity and greater inclusion of women in research projects, or, on the contrary, for specific studies on women. I believe that we have been slow to reflect on this reality, since female gender has been considered a predictor of mortality in CABG since the publication of CASS in 1981^4^ and has remained constant, including in our setting as part of InsCor.^5^ This was once again recognized in this important article by Goncharov et al.^6^ Identifying female sex as a predictor of mortality in a single-center and retrospective study with 9,845 patients, where less than 20% were women. However, unlike other studies, the difference in mortality between women and men was lost after pairing.^6^

The propensity score adjusts an analysis based on the observed variables, while randomized studies balance both known and unknown variables. Bias arises when differences in results may be a consequence of secondary characteristics of the patients, and this can be noticeable in a sample spanning almost 3 decades.^6^ Within this, perhaps the variable that may have most influenced these results was the inclusion of surgeries combined with CABG and not just CABG alone. Women in general are operated on with more comorbidities and arrive at a more advanced stage for surgery.

This is a global finding that may be related to the silent progression of heart disease in women or even have a behavioral and cultural component, which justifies the pairing of the groups. However, regardless of Goncharov having shown that after pairing in a retrospective single-center database, there was no difference in mortality between women and men, he concludes that female sex is indeed an independent predictor of mortality after CABG.^6^ A predictor that carries the influence of other known and unknown variables in our daily practice and that we must elucidate urgently^1,7,8^

For example, data show that in CABG, even though women have shorter surgery times, extracorporeal circulation, anoxia, and less use of arterial grafts and fewer distal anastomoses, they develop more immediate postoperative complications. This makes us reflect that we should not only standardize equal care, but that we should move towards personalized and optimized surgical care for women, both in diagnostic criteria and in preparation, and the best time for intervention, as well as in the best surgical strategies for each patient.^9^

Personalization is important; there are risk factors that are truly irreversible in women, such as a smaller physical constitution, as well as a smaller diameter and greater tortuosity of the coronary arteries, in addition to a higher prevalence of fragility. Hormonal variations make atherosclerotic disease more serious in women. Recent evidence shows that smaller physical size in women leads to greater hemodilution and lower hematocrit rates during cardiopulmonary bypass, justifying the need for more liberal levels of transfusion compared to men.^9,10^

One of the areas that has concerned us the most is related to the timing of surgical indication and the preparation of female patients, since the recommendations come from guidelines with an insignificant number of women.^11^ Therefore, we are releasing female patients for surgery with the same criteria as male patients, that is, with the same levels of serum hemoglobin, glycosylated hemoglobin, renal function, albumin, etc., which could explain the difference in results. This broadens our horizons, not only by seeking how to indicate better but also how to better prepare, treat, and care for women.^12^

Goncharov et al. are to be congratulated for being able to bring more discussion about the results of CABG in women, which reinforces the "Call to action" for more scientific evidence, including women, so that it can be used effectively in women.^6^ Promoting better care for women becomes a social necessity that brings benefits not only because they are the fastest growing population in the world, but also because it promotes the principle of inclusion and adapts to the specific needs that meet current policies. The phrase "Nothing about us without us is for us" is based on the principle of participation; it is used to communicate the idea that no policy should be decided without the full and direct participation of the members of the group(s) affected by it (Figure 1).^13,14^

**Figure 1:** Nothing about us without us is for us. Ricardo Levins Morales, 2010. Digital collections Joseph P. Healey Library.
